# Monthly Summary

**Published:** 1884-03

**Authors:** 


### MONTHLY SUMMARY.

---

CARIES DENTIUM.—First. The contact of saliva with amylaceous or saccharine food, (not to speak of nitrogenous food,) or a solution of sugar or starch in saliva, kept at body temperature, invariably gives rise in four or five hours to a strong acid reaction, due to the generation of an organic acid.

Second. There must consequently be in the human mouth a constant though variable generation of acid, because of the impossibility of keeping the mouth perfectly free from food and from solutions of amyloids in saliva, which penetrate cracks, pits, and fissures, or are held by capillary attraction between the surfaces of teeth in contact, and there become acid by fermentation.

Third. The degree of acidity depends somewhat upon the length of time which has elapsed since partaking of food, and will be found greatest on rising in the morning.

Fourth. A cavity of decay in which saccharine or amylaceous food has remained for some hours, must and will be found, always and without exception, to have an acid reaction.

Fifth. The extent to which any tooth suffers from the action of the acid depends upon its density and structure, but more particularly upon the perfection of the enamel and the protection of the neck of the tooth by healthy gums. What we might call the perfect tooth would resist indefinitely the same acid to which a tooth of opposite character would succumb in a few weeks.

Sixth. An occasional possible absence of an acid reaction in a cavity of decay is no indication that acid has not participated in the production of the cavity. Little or no value can be attached to tests of the saliva alone.

Seventh. Any general or special disorder or condition of the system which results in the withdrawal of lime salts from a tooth, or in a lowering of its density, or in a weakening of the chemical union between the organic and inorganic matter of the tooth, renders it more liable to decay.

Eighth. Strong acid and corroding substances brought but momentarily into the human mouth, may give rise to lesions of the enamel at points where the ordinary agents alone could never have begun.

Ninth. All the *macros*copical appearances and characteristics of caries may be produced with the greatest exactness *out* of the mouth, simply by subjecting teeth to those acid mixtures which are constantly to be found *in* the mouth.

Tenth. The superficial layers of carious dentine undergo an almost, if not absolutely complete decalcification, which decreases as we approach the normal dentine. The same is true of dentine decalcified in saliva and bread.

Eleventh. The destruction of the organic constitutents follows (not precedes) the decalcification, and is evidently for the most part to be ascribed to the action of the fungi.

Twelfth. The fungi found in the human mouth do not participate *directly* in the process of decalcification. The exact part which they perform in the production of an acid reaction requires further investigation.

Thirteenth. The fungi produce the most manifold anatomical changes in the softened dentine, resulting in the complete obliteration of the structure and final disappearance of the tissue in a mass of debris and fungi.

Fourteenth. The invasion of the micro-organisms is always preceded by the extraction of the lime salts.

Fifteenth. The destruction of the tissue remaining after decalcification is effected almost wholly by fungi alone.

Sixteenth. Inflammation can hardly be looked upon as a very important factor in caries of the teeth.

Seventeenth. Caries of the enamel is purely chemical, the decalcification resulting at once in the complete dissolution of the tissue.

Eighteenth. Caries of cement runs a course analogous to caries of dentine, a softening of the tissue by acids, and following this its destruction by fungi; a slight inflammatory action on the part of the living matter in the corpuscles, is not to be excluded.—MILLER, *Independent Practitioner.*

---

BEST METHOD OF PRODUCING ANÆSTHESIA.—First decide whether the person is in condition to take an anæsthetic. If there is neither heart trouble nor other obstacle in the way, let the patient fast about six hours previous to the administration.

Have ready the following:

1st.—Alcohol........................................1 ounce.
Chloroform (Squibs)........................2 ounces.
Ether (Squibs)........... ...................3 ounces.

M

S. Anæsthetic.

2d.—A bag made by placing a newspaper on a towel or napkin, and folded in such a way as to cover the chin and nose. Let it be about five inches deep. Fasten a piece of wadding to the bottom of the bag to receive the anæsthetic.

3d.—A little cotton in a small vial with a few drops of nitrate of amyl on it.

4th.—An empty basin to hold a big sponge or towel, and near by some hot water.

5th.—The clothes of the patient perfectly loose, even to the garters, so that nothing may impede circulation; and see to it that the region of the heart may be easily reached.

Now why all these preparations? I can answer best by describing a case, the only one which might have resulted seriously.

I had to perform an operation. The assistant who administered the anæsthetic, forgot to "mind his own business;" he was anxious to see what I was doing. All at oncc he exclaimed: "Her eyes are wide open; she doesn't breathe!"

Now what? Be cool, for then you will do what is right, and do it quickly and well, which are all important, for life now hangs by seconds.

Quicker than I can tell it, I flung away the instrument I was holding, put my left hand under the shoulder of the patient, pulled her out of bed in such a manner that her buttocks rested on the bed and her head touched the floor, and called out "hot water!" I bared the breast and pressed the hot-water-soaked towel to her heart, and then—a gasp.

The lowering of the head alone might have answered; the hot water to the heart was certainly a great help, and had the breathing not been re-established immediately, I would have ordered the nitrate of amyle held to the nose.

How very important is it that even the minutest things be thought of and be prepared before hand. Be prepared for emergencies always!—*Medical Truth.*

